# Effects of resistance training on patients with End-Stage Renal Disease: an umbrella review with meta-analysis of the pooled findings

**DOI:** 10.1007/s40620-023-01635-7

**Published:** 2023-06-15

**Authors:** Borja Perez-Dominguez, Luis Suso-Marti, Fernando Dominguez-Navarro, Sara Perpiña-Martinez, Joaquin Calatayud, Jose Casaña

**Affiliations:** 1 Exercise Intervention for Health Research Group (EXINH-RG), Department of Physiotherapy, University of Valencia, 46010 Valencia, Spain; 2 Department of Nursing and Physiotherapy, Pontifical University of Salamanca, Salamanca, Spain

**Keywords:** Dialysis, Exercise, Meta-analysis, Resistance training

## Abstract

**Objectives:**

This umbrella review aimed to review the effects of resistance training on patients with end-stage renal disease and assess the methodological quality of the available literature.

**Methods:**

An umbrella review and meta-meta-analysis was performed. A systematic search was conducted until May 2022. Article selection, quality assessment, and risk of bias assessment were performed by two independent reviewers. The meta-meta-analyses were performed with a random-effects model and the summary statistics were presented in the form of a forest plot with a weighted compilation of all standardized mean differences and corresponding 95% confidence interval. Twenty-four reviews were eventually included. The protocol was registered in the international registry PROSPERO (CRD42022321702).

**Results:**

Resistance training showed positive effects on functional capacity (*g* = 0.614), aerobic capacity (*g* = 0.587), health-related quality of life (*g* = 0.429), and peak force (*g* = 0.621). Fifteen of the included studies (63%) presented low risk of bias, and the remaining studies (37%) showed unclear risk of bias.

**Conclusion:**

Resistance training in patients undergoing hemodialysis is an intervention that shows positive results regarding physical and functional outcomes. The quality level of the literature is inconclusive, but the included studies present low risk of bias.

**Graphical abstract:**

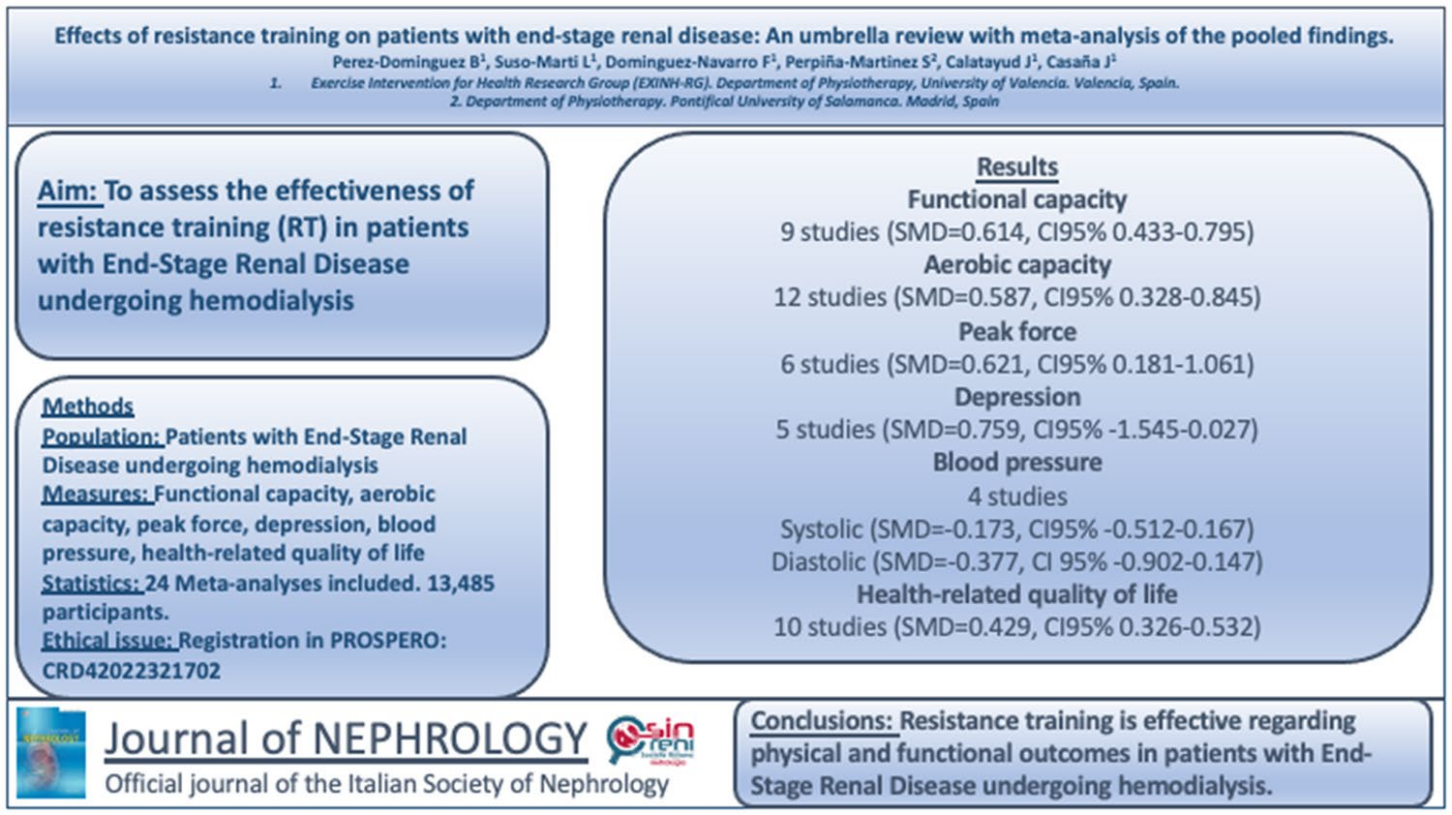

## Introduction

End-Stage Renal Disease (ESRD) is an increasing worldwide concern that involves significant healthcare costs [1]. Global estimates reveal 8–16% prevalence rates that are progressively growing and will end up presenting a major challenge for healthcare systems [2].

Among the possible treatment options, most patients suffering from ESRD receive hemodialysis [3], a treatment that is often related to several impairments, such as decreased functional capacity, decreased levels of physical activity and increased mortality rates [4]. The most significant predictor of mortality rates in this population is skeletal muscle wasting [5, 6]. This is very common in patients with ESRD due to sedentary behavior [7], acidosis, comorbid illnesses, corticosteroid usage, aging, oxidative stress, insulin resistance, chronic inflammation and problems related to restrictions in dietary protein intake [8]. All these factors lead to loss of muscle strength and the development of functional impairments [7, 9].

To revert and prevent the development of skeletal muscle wasting, exercise has been extensively introduced as a complement to daily dialysis care routines. Exercise modalities include aerobic, resistance and combined programs, which are delivered during dialysis sessions or on non-dialysis days. Among them, resistance training has become a well-established, safe, and effective modality to overcome skeletal muscle wasting [10, 11], being the choice of preference of many authors when implementing exercise sessions during dialysis.

Several resistance training programs [10], such as the “Progressive Exercise for Anabolism in Kidney Disease (PEAK)” or clinical exercise guidelines such as the one developed by The Life Options Rehabilitation Advisory Council (LORAC), “Exercise. A guide for people on dialysis” have also been created to support these implementations [12]. To better understand the benefits and the effects of resistance training in patients with ESRD, several clinical trials, and further systematic reviews and meta-analyses, have been conducted. However, to the best of our knowledge, there is no review that involves the assessment of the overall effect and quality of these studies, which could help to establish guidelines and recommendations when implementing resistance training in these patients. Therefore, our research team conducted an umbrella review based on the evidence found involving resistance training in ESRD patients. This study aims to provide an overview of the benefits of resistance training in patients suffering from ESRD and an assessment of the quality of the published literature.

## Methods

### Design

A systematic review of reviews was conducted in accordance with the Preferred Reporting Items for Overviews of Systematic Reviews including harm checklist (PRIO-harms), which consists of 27 items and 56 sub-items, followed by a 5-stage process flow diagram [13]. The protocol was registered in the international registry PROSPERO (CRD42022321702).

### Inclusion criteria

The criteria used in this search involved methodological and clinical outcome factors, including population, intervention, comparison, and study type (PICOS) criteria [14].

Participants (population) included in this search were adult (> 18 years old) patients that suffered from CKD who were undergoing maintenance hemodialysis. The trial (intervention) had to include either a resistance exercise training program or a combined exercise program that included resistance exercises. Comparator groups (control) received no-exercise, sham, or placebo exercise interventions. Assessments (outcomes) involved functional capacity assessed through the 6-Minute Walk Test (6MWT), aerobic capacity assessed through maximum oxygen intake (VO_2max_), peak force assessed through dynamometry, health-related quality of life (HRQOL) assessed through the Short-Form 36 (SF-36) questionnaire, depression symptoms assessed through the Beck Depression Inventory (BDI), and changes in systolic and diastolic blood pressures. Papers included systematic reviews with a meta-analysis of randomized controlled, randomized uncontrolled or controlled clinical trials.

### Search strategy

A systematic search was carried out in the following scientific databases for related papers published by May 27, 2022: MEDLINE (Pubmed), The Cochrane Library (CENTRAL), EMBASE, CINAHL, PEDro and Google Scholar. The search strategy combined medical subject headings (MeSH) through Boolean operators “OR” and/or “AND”. The following headings were applied: “hemodialysis”, “renal dialysis”, “dialysis” “kidney failure, chronic”, “renal insufficiency, chronic”, “strength training” and “resistance training”.

Two independent researchers (BPD and LSM) carried out the search strategy following the same methods, and differences were resolved by consensus, mediated through a third researcher (JCG). Researchers manually searched journals that had published related papers as well as the reference lists of the included papers. Duplicates were hand-checked manually before being removed.

### Selection criteria and data extraction

Two independent researchers conducted a data analysis to assess the relevance of the reviews according to the search question. Initially, this analysis was carried out by extracting information regarding the title, abstract and keywords of the systematic reviews. If there was no consensus between the researchers or if the title and abstract contained insufficient information, the full text was reviewed. After this first analysis, the researchers screened the reviews to assess whether they met the inclusion criteria. Differences between researchers were resolved through discussion and mediation by a third researcher.

The extraction protocol was based on the Cochrane Review Groups: (1) study characteristics, including author, title, and year of publication, (2) review information, including sample size, patients, and types of trials, (3) description of the intervention and control, including types of exercise, parameters of frequency, intensity and time, and study outcomes such as functional capacity, aerobic capacity, strength, health-related quality of life, depression symptoms, and blood markers.

### Risk of bias analysis

Two independent researchers (BPD and LSM) assessed the quality of the included reviews by analyzing their risk of bias through the Risk of Bias in Systematic Reviews (ROBIS) tool [15]. This tool assesses quality across four domains: (1) study eligibility, (2) study identification and selection, (3) data collection and study appraisal and (4) synthesis of findings. ROBIS provides an overall assessment of high, low, or unclear risk of bias. Both researchers assessed the quality of the systematic reviews using the same method. Disagreements between the researchers were resolved by consensus mediated by a third researcher. Interrater reliability was determined using the kappa coefficient (κ), using κ > 0.7 to indicate a high level of agreement; κ = 0.5–0.7 to indicate a moderate level of agreement; and κ < 0.5 to indicate a low level of agreement [16].

### Statistical analysis

Statistical analysis was performed using the “metaumbrella” app and its associated package in R, which is a tool that allows users to perform umbrella reviews with stratification of evidence [17]. Meta-analytical procedures were carried out following recommendations on how to conduct umbrella reviews [18]. We used the same inclusion criteria for the systematic review and meta-analysis but added two criteria: (1) The Results section contained detailed information on the comparative statistical data (mean, standard deviation, and/or 95% confidence interval [CI]) of the main variables, and (2) data for the analyzed variables were represented in at least three meta-analyses. Common effect sizes were estimated using standardized mean differences (SMDs), assessed through Hedge’s *g* to compare continuous variables, and their 95% confidence intervals using a random-effects model. The heterogeneity of each meta-analysis was estimated using the *I*-squared statistic (*I*^2^), establishing an *I*^2^ of 25% to represent a small degree of heterogeneity, 50% a moderate degree and 75% a large degree [19]. To obtain a pooled estimate of the effect in the meta-analysis of the heterogeneous studies, we performed a random-effects model, as described by DerSimonian and Laird [20]. The estimated SMDs were interpreted as described by Hopkins et al. an SMD of 4.0 was considered to represent an extremely large clinical effect; 2.0–4.0 a very large effect; 1.2–2.0 a large effect; 0.6–1.2 a moderate effect; 0.2–0.6 a small effect; and 0.0–0.2 a trivial effect [21].

### Overall strength of the evidence

Strength of the evidence across the systematic reviews was assessed using the Physical Activity Guidelines Advisory Committee (PAGAC) [22]. For the PAGAC analysis, the findings were evaluated according to five criteria: (1) applicability of the study sample, exposures, and outcomes to the research question; (2) generalizability to the population of interest; (3) risk of bias or study limitations; (4) quantity and consistency of findings across studies; and (5) magnitude and precision of the effect. The strength of the evidence was classified according to the PAGAC as strong, moderate, limited or not assignable.

### Overlapping analysis

To assess the overlap of primary studies among systematic reviews the GROOVE (Graphical Representation of Overlap for OVErviews) methodological approach was employed. This tool is intended to be used mainly by authors of overviews of systematic reviews. The tool was made in Microsoft Excel, which we think could boost its wide usage [23].

## Results

**Fig. 1:**
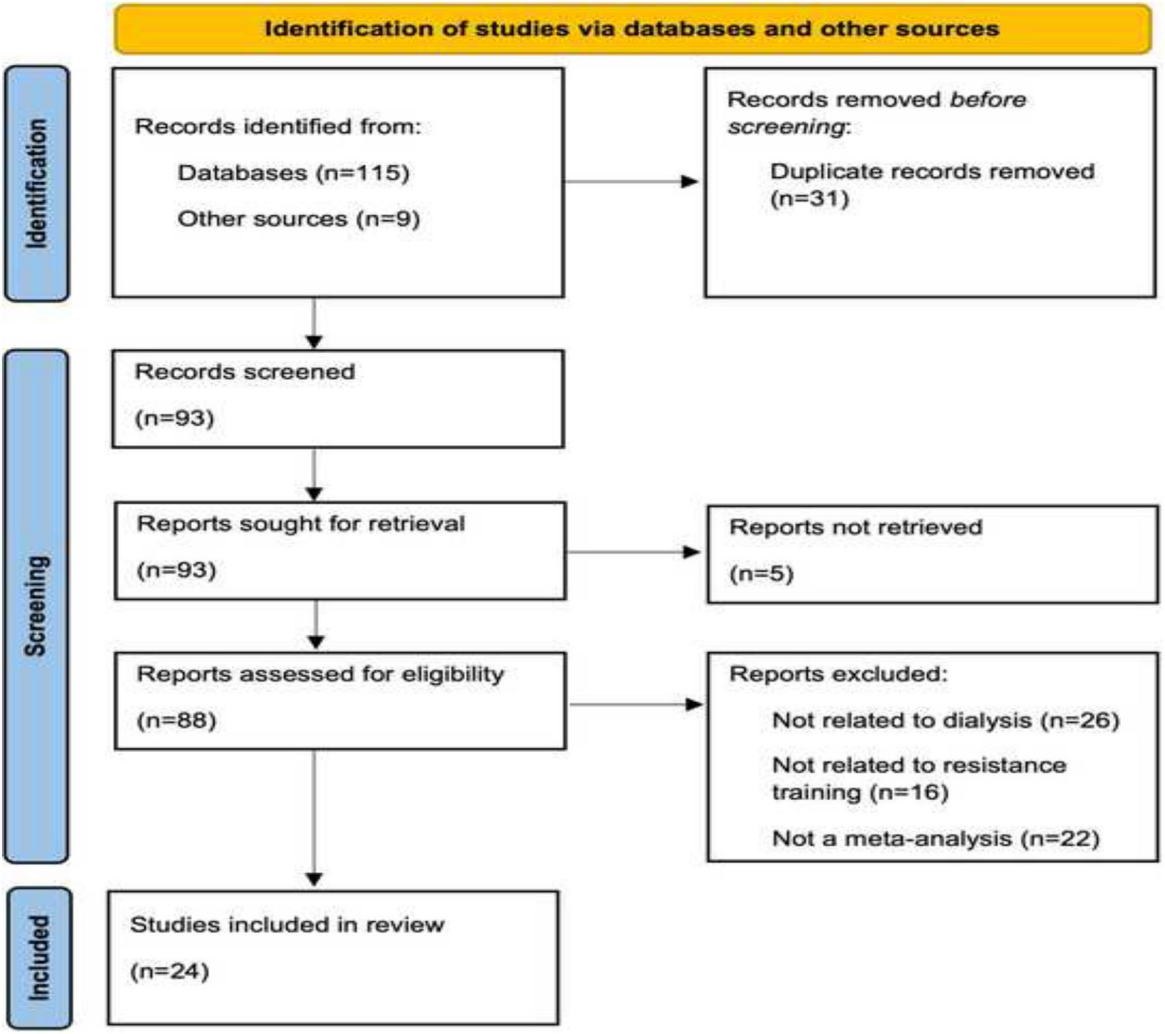
PRISMA flow Diagram

Figure 1 presents the PRISMA flow diagram, showing the different stages of the review process, including reasons for exclusion. One hundred fifteen initial studies were identified through databases and nine additional studies were identified through other sources. After removing duplicates and eliminating studies that the authors were unable to retrieve, 88 studies were screened for eligibility. Studies were excluded if they were not related to a population of patients suffering from ESRD that underwent treatment with dialysis or if the intervention did not include a form of resistance training. After excluding all the papers that did not meet the criteria, 24 studies were finally included in the analysis.

### Characteristics of the included studies

Table 1 reports the characteristics of the studies included in the umbrella analysis (*n* = 24). Studies were published between 2010 and 2021 and included a total of 13,415 participants. Participants included in the studies were adults (> 18 years) suffering from ESRD under maintenance dialysis, who received an intervention of either a resistance training program or a combined training program that included resistance exercises. Specific details of the included interventions are summarized in Table 2. Exercise interventions lasted from 4 weeks to 1 year, exercise was mostly performed 2–3 times per week and lasted from 10 min to 2 h. Exercise intensity was mainly assessed with Borg’s Rating of Perceived Exertion scale. Control or comparison groups were defined as patient’s receiving regular dialysis treatment, considered to be usual care, or placebo or sham exercise, that consisted in stretching exercises or passive range of movement exercises.

**Table 1:** Description of meta-analysis studies included

Study	Number of studies	Design of studies	Sample in RT interventions	CKD Type and patient characteristics	Intervention and control group	Outcomes		
						Number of RT studies included in meta-analysis	Scales of measurement	Results
Andrade et al. [24]	15	RCTs	*n* = 243	CKD patients who underwent intradialytic exercise protocols *Mean age range:* 42–68 years	*Intervention:* RT + AE or RT + AE + Flexibility *Control:* RDT	5	Functional aerobic capacity (VO_2max_)	Significant improvements (MD = −1.64, CI 95% −3.21 to −0.07)
Bernier-Jean et al. [25]	89	RCTs and quasi-RCTs (allocation to treatment was obtained through alternation)	*n* = 1.245	Adults receiving maintenance dialysis treatment *Mean age range*: 30–72 years	*Intervention*: RT, RT + AE or Pilates *Control*: No exercise or placebo exercise	36	Functional capacity (6MWT)	Improved scores RT (MD: 44.7, CI 95% 27.0–62.4) Improved scores RT + AE (MD = 53.6, CI 95% 39.4–67.9)
							HRQOL (SF-36)	Improved scores RT (MD = 2.5, CI 95% 1.3–6.3) Improved scores RT + AE (MD = 4.4, CI 95% 1.9–6.8)
							HRQOL (SF-36)	Improved scores RT (MD = 0.7, CI 95% −5.9 to 4.6) Improved scores RT + AE (MD = 2.6, CI 95% 1.7–6.9)
							HRQOL (SF-36)	Improved scores RT (MD = 10.7, CI 95% 6.5–28.0) Improved scores RT + AE (MD = 4.0, CI 95% 2.5–10.5)
							Depression (BDI)	Improved scores RT (SMD = 0.52, CI 95% 0.92 to 0.12) Improved scores RT + AE (SMD = -1.0, CI 95% -1.7 to -0.3)
Bogataj et al. [26]	104	RCTs with a pre-post intervention lasting > 8 weeks	*n* = 909	Adult end-stage kidney disease HD patients *Average age*: 55.7 ± 9.6 years	*Intervention:* RT or RT + AE *Control:* Usual care	20	Functional Capacity (6MWT)	Moderate effects RT (ES = 0.10, CI 95% −0.19 to 0.39) Moderate effects RT + AE (ES = 0.71, CI 95% −0.06 to 1.48)
							Functional aerobic capacity (VO_2max_)	Moderate effects RT + AE (ES = 0.67, CI 95% 0.33–1.01)
Cheema et al. [27]	23	RCTs	*n* = 271	Adults with CKD (Stage 1–5) receiving maintenance HD *Mean age range*: 43–69 years	*Intervention*: RT *Control*: Usual care or stretching exercises	7	Peak force (dynamometry)	Significant improvement RT (SMD = 1.15, CI 95% 0.80–1.49)
							HRQOL (SF-36)	Significant improvement RT (SMD = 0.83, CI 95% 0.51–1.16)
Chung et al. [28]	17	RCTs	*n* = 284	Participants aged > 18 years with ESRD requiring HD > 3 months *Mean age range*: Not reported	*Intervention*: RT, RT + AE *Control*: No exercise	5	Functional aerobic capacity (VO_2max_)	Significant improvement RT + AE (SMD = 0.50, CI 95% 0.26–0.74)
							Depression (BDI)	Significant improvement RT + AE (SMD = −0.80, CI 95% −1.10 to −0.50)
							HRQOL (SF-36)	Significant improvement RT + AE (SMD = 0.46, CI 95% 0.20–0.73)
Clarkson et al. [29]	27	RCTs	*n* = 616	Patients aged $\ge 18$ years with stage 5 CKD undergoing HD *Mean age range*: Not reported	*Intervention*: RT, RT + AE, EMS *Control*: Usual care, non-exercising or undergoing only range of motion passive exercises	15	Functional capacity (6MWT)	Significant improvement RT (ES = 23.62, CI 95% 6.45–40.79)
Ferrari et al. [30]	50	RCTs	*n* = 782	Adults $\ge 18$ years with ESRD undergoing HD *Mean age range*: 20–73.9 years	*Intervention*: RT, RT + AE *Control*: Usual care or sham exercises	20	Functional capacity (6MWT)	Significant improvement RT (MD = 68.50, CI 95% 29.05–107.96)
							Functional aerobic capacity (VO_2max_)	Significant improvement RT + AE (MD = 5.41, CI 95% 4.03–6.79)
							Diastolic BP (mmHg)	Significant reduction RT + AE (MD = −5.76, CI 95% −8.83 to −2.70)
Ferreira et al. [31]	8	RCTs	*n* = 179	Adults > 18 years with CKD on maintenance hemodialysis *Mean age range*: 18–70 years	*Intervention*: RT + AE *Control*: Routine care or education exercise	4	Depression (BDI)	Significant improvement RT + AE (SMD = −0.66, CI 95% −1.00 to −0.33)
Gomes Neto et al. [32]	56	RCTs	*n* = 1.255	Maintenance HD patients *Mean age range*: 20–73.9 years	*Intervention*: RT, RT + AE *Control*: No exercise	27	Functional aerobic capacity (VO_2max_)	Significant improvement RT + AE (MD = 5.1, CI 95% 3.4–6.8)
							Functional capacity (6MWT)	Significant improvement RT (MD = 30.2, CI 95% 24.6–35.9) Significant improvement RT + AE (MD = 45.2, CI 95% 7.8–82.6)
							Peak force (dynamometry)	Significant improvement RT (MD = 12.5, CI 95% 0.9–24.0)
							HRQOL (SF-36)	Significant improvement RT Physical Component Score (MD = 9.53, CI 95% −3.09 to 22.15) Significant improvement RT + AE Physical Functioning (MD = 10.67, CI 95% 1.08–20.25) Significant improvement RT + AE Vitality (MD = 10.01, CI 95% 4.30–15.72)
							Depression (BDI)	Significant reduction RT + AE (MD = −7.3, CI 95% −9.3 to −5.3)
Heiwe et al. [33]	41	RCTs	*n* = 787	Adults with CKD (Stage 2–5) *Mean age range*: 36–71 years	*Intervention*: RT, RT + AE *Control*: No exercise or sham exercise	18	Functional aerobic capacity (VO_2max_)	Significant improvement RT + AE (SMD = −0.77, CI 95% −1.06 to −0.48)
							Diastolic BP (mmHg)	Significant improvement RT + AE (SMD = 0.41, CI 95% 0.02–0.80)
							Systolic BP (mmHg)	Significant improvement RT + AE (SMD = 0.33, CI 95% 0.04–0.62)
							Peak force (dynamometry)	Significant improvement RT + AE (SMD = −0.43, CI 95% −0.79 to −0.08) Significant improvement RT (SMD = −0.55, CI 95% −0.90 to −0.19)
Hu et al. [34]	33	RCTs	*n* = 992	Adults $\ge$ 18 years diagnosed with ESRD under HD > 3 months *Mean age range*: Not reported	*Intervention*: RT, RT + AE *Control*: Usual care, sham exercise, or no exercise	21	HRQOL (SF-36)	Significant improvement RT Physical Component Scale (SMD = 0.23, CI 95% 0.02–0.44) Significant improvement RT + AE Physical Component Scale (SMD = 0.50, CI 95% 0.22–0.77) Significant improvement RT Mental Component Scale (SMD = 0.28, CI 95% 0.03–0.54) Significant improvement RT Physical Functioning (SMD = 0.53, CI 95% 0.16–0.90) Significant improvement RT + AE Physical Functioning (SMD = 0.49, CI 95% 0.05–0.92) Significant improvement RT + AE Role Physical (SMD = 0.21, CI 95% −0.09–0.50) Significant improvement RT Vitality (SMD = 0.37, CI 95% 0.12–0.63) Significant improvement RT + AE Role Emotional (SMD = 0.34, CI 95% 0.04–0.64) Significant improvement RT Mental Health (SMD = 0.44, CI 95% 0.13–0.74)
Huang et al. [35]	20	RCTs	*n* = 435	Participants > 18 years diagnosed with ESRD under HD > 3 months *Mean age range*: 30–70 years old	*Intervention*: RT, RT + AE *Control*: Usual care or sham exercise	10	Functional aerobic capacity (VO_2max_)	Significant improvement RT + AE (SMD = 0.78, CI 95% 0.51–1.05)
Lu et al. [36]	21	RCTs	*n* = 539	Adult participants (age > 18 years old) with clinical diagnosis of kidney failure undergoing HD > 3 months *Mean age range*: Not reported	*Intervention*: RT, RT + AE *Control*: Usual care, no exercise, sham exercise, attention care or placebo	14	Physical performance (6MWT)	Significant improvement RT (MD = 41.92, CI 95% 8.06–75.75)
							Peak force (dynamometry)	Significant improvement RT (MD = 3.93, CI 95% 0.59–7.28)
Matsuzawa et al. [37]	39	RCTs	*n* = 759	Participants at least 18 years of age undergoing hemodialysis *Mean age range*: Not reported	*Intervention:* RT, RT + AE *Control:* Usual care or no exercise	19	Physical performance (6MWT)	Significant improvement RT + AE (SMD = 0.58, CI 95% 0.24–0.93)
							Functional aerobic capacity (VO_2max_)	Significant improvement RT + AE (SMD = 0.62, CI 95% 0.38–0.87)
							Peak force (dynamometry)	Significant improvement RT + AE (SMD = 0.94, CI 95% 0.67–1.21)
							HRQOL (SF-36)	Significant improvement RT + AE (SMD = 0.53, CI 95% 0.52–0.82)
Molsted et al. [38]	8	RCTs	*n* = 290	Participants $\ge$ 18 years undergoing HD $\ge$ 1 month *Mean age range*: 40.2–71.1 years	*Intervention*: RT *Control*: Usual care or stretching	8	HRQOL (SF-36)	Significant improvement RT Physical Component Scale (MD = 10.05, CI 95% 2.95–17.14) Significant improvement RT Physical Function (MD = 9.38, CI 95% 0.79–17.97)
Pu et al. [39]	27	RCTs	*n* = 499	Adult patients on HD > 3 months *Mean age*: 53 years	*Intervention:* RT, RT + AE *Control*: No exercise	11	Functional aerobic capacity (VO_2max_)	Significant improvement RT + AE (MD = 4.11, CI 95% 2.94–5.27)
							Depression (BDI)	Significant reduction RT + AE (SMD = −1.16, CI 95% −1.86 to −0.45)
							HRQOL (SF-36)	Significant improvement RT + AE (MD = 7.72, CI 95% 1.93–13.51)
							Diastolic BP (mmHg)	Significant improvement RT + AE (MD = −4.11, CI 95% −6.50 to −1.72)
							Systolic BP (mmHg)	Significant improvement RT + AE (MD = −4.87, CI 95% −9.20 to −0.55)
Scapini et al. [40]	33	Controlled trials	*n* = 782	Adults requiring HD for ESRD *Mean age range*: Not reported	*Intervention*: RT, RT + AE *Control*: No exercise or placebo	15	Functional aerobic capacity (VO_2max_)	Significant improvement RT + AE (MD = 5.00, CI 95% 3.50–6.50)
							Diastolic BP (mmHg)	Significant reduction RT + AE (MD = −5, CI 95% −6 to −3)
							Systolic BP (mmHg)	Significant reduction RT + AE (MD = −9, CI 95% −13 to −4)
Schardong et al. [41]	10	RCTs and controlled clinical trials	*n* = 224	Patients with CKD undergoing HD > 3 months *Mean age range*: 46.4–68.6 years	*Intervention*: RT *Control*: Control, placebo, or exercise with no EMS	8	Peak force (dynamometry)	Significant improvement RT Lower limb (SMD = 1.46, CI 95% 0.86–2.07) Significant improvement RT Upper limb (MD = 10.02, CI 95% 0.78–19.27)
							Functional capacity (6MWT)	Significant improvement RT (MD = 30.11, CI 95% 15.57–44.65)
Segura-Orti [42]	14	RCTs	*n* = 364	Adults (> 18 years) undergoing HD *Mean age range*: 32–65 years	*Intervention*: RT, RT + AE *Control*: No exercise or placebo	6	HRQOL (SF-36)	Significant improvement RT Physical Component Scale (SMD = 11.03, CI 95% 5.63–16.43)
							Functional aerobic capacity (VO_2max_)	Significant improvement RT + AE (SMD = 5.57, CI 95% 2.52–8.61)
Sheng et al. [43]	24	RCTs	*n* = 451	HD patients *Mean age range*: 46.6–69 years	*Intervention*: RT, RT + AE *Control*: No exercise	11	Functional aerobic capacity (VO_2max_)	Significant improvement RT + AE (SMD = 0.92, CI 95% 0.53–1.31)
Wu et al. [44]	12	RCTs and quasi-randomized controlled trials	*n* = 745	Adults $\ge$ 18 years with CKD stages 1–5 *Mean age range*: 47.5–80.3 years	*Intervention*: RT + AE *Control*: Usual care and no exercise	12	Diastolic BP (mmHg)	Significant improvement RT + AE (MD = −3.63, CI 95% −5.35 to −1.91)
							Systolic BP (mmHg)	Significant improvement RT + AE (MD = −5.24, CI 95% −7.93 to −2.54)
Zhang et al. [45]	14	RCTs	*n* = 594	Adults (> 18 years) undergoing HD > 3 months *Mean age range*: 28.5 to 81.4 years	*Intervention*: RT *Control*: No exercise or stretching	14	Functional capacity (6MWT)	Significant improvement RT (SMD = 0.52, CI 95% 0.28–0.75)

*ADLSS* activity daily living summary score; *AE* aerobic exercise; *BAI* Beck Anxiety Index; *BALP* bone-specific alkaline phosphatase; *BDI* Beck depression inventory; *BMD* bone mineral density; *BP* blood pressure; *CI* confidence interval; *CKD* chronic kidney disease; *CPET* cardiopulmonary exercise test; *CRP* C-reactive protein; *CSA* cross sectional area; *DASS* Depression Anxiety Stress Scale; *DBS* diastolic blood pressure; *EMS* electrical myostimulation; *ES* effect size; *GXT* graded exercise test; *ESRD* end stage renal disease; *HADS* Hospital Anxiety Depression Scale; *HD* hemodialysis; *HRQOL* health-related quality of life; *L* left; *LBM* lean body mass; *MD* mean difference; *MRI* magnetic resonance imaging; *OPG* osteoprotegerin; *PASE* physical activity scale elderly; *RCT* randomized controlled trial; *RDT* regular dialysis treatment; *R* right; *RT* resistance training; *SBP* systolic blood pressure; *SD* standard deviation; *SF-36* short-form 36 questionnaire; *STS* sit-to stand-to sit test; *6MWT* 6 Minute Walking Test

**Table 2:** Interventions included in each of the systematic reviews

Study	Group	Exercise modality	Intervention characteristics			Intervention duration	Frequency	Session duration
			RT Protocol (distribution and exercise type)	RT Work interval (duration and intensity)	RT Rest interval (duration and intensity)			
Andrade et al. [24]	Exercise	2 RT + AE, 3 RT + AE + Flexibility	- Prior to dialysis or during dialysis - 2–10 min WU - From 2 sets × 8 repetitions to 3 sets × 15 repetitions - Exercises with *thera-bands* and weights - Isotonic and isometric exercises for abdomen and lower limbs	- Not reported - Intensity Borg scale (RPE) 13 or 60–70% of the HRMAX	Not reported	3–12 months	Three times per week	30–90 min or not reported
	Control	RDP						
Bernier-Jean et al. [25]	Exercise	21 RT, 19 RT + AE	-Prior to dialysis or during dialysis - 2–10 min WU - From 1 to 4 sets × 8 to 30 repetitions - Solely lower body exercises or both upper and lower limb exercises - Closed and open chain strengthening exercises - Weights, resistance bands, both, Response Seated Leg Curl Thigh Extension pulley or leg press machine	- Concentric phase as fast as possible - Concentric phase 1.5 s, isometric phase 0.5 s and eccentric phase 3 s - 5 s isometric contraction - Intensity Borg scale (RPE), Omni scale, % of 1, 3 or 5RM, 60–70% of the HRMAX - Not reported	- 1–2-min rest between sets - 1 min between sets, 3 min between exercises - 3-min rest between exercise periods - According to the necessity of the patient - Not reported	8 weeks to 2 years	2–5 times per week	30–110 min or not reported
	Control	No exercise or placebo exercise						
Bogataj et al. [26]	Exercise	9 RT, 12 RT + AE	- Prior to dialysis or during dialysis - 2–10 min WU - From 2 to 3 sets × 8 to 10 repetitions - Free-weight dumbbells and weighted ankle cuffs - Equipment designed to fit the end of the dialysis chair - Response Seated Leg Curl Thigh Extension pulley - Calisthenics, steps and low weight exercises	- 4:1 work/rest intervals or not reported - Intensity Borg scale (RPE), % of the 1-5RM, 50–60% of VO_2max_, 60–70% of HR_max_ or subjective assessment	- 1–2 min between sets and exercise - Not reported	8–40 weeks	2–4 times per week	13–90 min or not reported
	Control	Usual care, stretching, walking program at home or ROM exercises						
Cheema et al. [27]	Exercise	7 RT	- Prior to dialysis or during dialysis - From 2 to 3 sets × 8 to 15 repetitions - Both upper and lower limb exercises - Exercise with weights, elastic bands or sandbags - Exercise using weighted dumbbells, ankle cuffs and elastic tubing - Standard machine weights -Pneumatic resistance equipment - Exercises based con the DPGE	- 1.5 s concentric phase, 0.5 s isometric and 3 s eccentric phase or not reported - Intensity 40–80% of 1-3RM or 15–17 Borg’s scale	1–2 min between sets or not reported	From 8 to 24 weeks	From 2 to 3 times per week	20–45 min or not reported
	Control	Usual care or stretching exercises						
Chung et al. [28]	Exercise	4 RT, 1 RT + AE	- During dialysis or immediately before - From 2 to 3 sets × 8 repetitions - Both upper and lower limbs - Exercise with ankle weights, *Thera-band* tubing or free-weight dumbbells - EMS	- 20 s stimulation/ 20 s rest - Not reported - Intensity 60% of 3RM or 15–17 Borg’s scale	- 1–2 min between sets and exercises - Not reported	From 8 to 20 weeks	From 2 to 3 times per week	10–60 min or not reported
	Control	No exercise						
Clarkson et al. [29]	Exercise	9 RT, 3 RT + AE, 3 EMS	- From 1 to 3 sets × 8 to 15 repetitions - Free-weight exercises with dumbbells for the upper body and ankle weights for the lower body - Sets of 10 to 12 exercises, combining upper body, lower body and core training	- From 2 to 20 s EMS stimulation - Not reported - Intensity % of 1–3 RM or 9–17 Borg’s scale	- 10–50 s rest between EMS stimulation - Not reported	From 8 to 22 weeks	From 2 to 3 times per week	15–60 min or not reported
	Control	Usual care, non-exercising or undergoing only range of motion passive exercises						
Ferrari et al. [30]	Exercise	14 RT, 7 RT + AE	- During dialysis or on non-dialysis days - 5-to-10-min WU - From 2 to 3 sets × 8 to 20 repetitions - Isometric and isotonic exercises for the abdomen and lower limbs - Participants asked to perform as many repetitions as possible - Exercises in both lower limbs and the non-dialyzed upper limb - Exercise with ankle weights, dumbbells and elastic bands - Leg press equipment	- 3–5 s isometric contraction - Co-isometric contraction held for 10 s - 2 s concentric and 2 s eccentric for each repetition - Not reported - Intensity 40–80% of the 1-3RM, according to participant’s subjective tolerance or 15–17 Borg’s scale	- 1-to-2-min rest between sets - 1 min rest between sets and 3-min rest between exercises - 1 min rest between sets and exercises - Rest according to the necessity of the participants - Not reported	From 8 weeks to 1 year	From 2 to 3 times per week	From 10 min to 2 h
	Control	Usual care or sham exercises						
Ferreira et al. [31]	Exercise	4 RT + AE	- During dialysis or non-dialysis days - From 2 to 3 sets × 10 to 15 repetitions - 7 exercises involving lower and upper limbs - Dynamic closed and open-chained exercises - Exercise with *Thera-band* resistive bands and soft weights	- Not reported - Intensity 50% of 1RM or 13–15 Borg’s scale	- Not reported	From 4 weeks to 1 year or not reported	3–4 times per week	From 15 to 90 min
	Control	*Control*: Routine care or education exercise						
Gomes Neto et al. [32]	Exercise	16 RT, 10 RT + AE	- Before, after or during dialysis - From 2 to 5-min WU - From 4 to 10 types of exercises - From 2 to 4 sets × 8 to 30 repetitions - Concentric and eccentric contractions - Isotonic and isometric exercises for lower limbs and abdomen - Exercises with free-weight dumbbells, thick colored elastic bands, ankle cuffs, sandbags and leg press equipment - Response Seated Leg Curl Thigh Extension pulley	- 1.5 s concentric phase, 0.5 s isometric and 3 s eccentric phase - 5 s isometric contraction - Co-isometric contraction held for 10 s - Not reported - Intensity 60–80% of 1-3RM, 11–17 Borg’s scale, according to participant’s tolerance or not reported	-1-to-2-min rest between sets and exercises - 3-min rest between exercises - 60 s rest between sets - Not reported	From 8 weeks to 1 year	From 2 to 3 times per week	From 20 to 90 min or not reported
	Control	No exercise						
Heiwe et al. [33]	Exercise	13 RT, 5 RT + AE	- During dialysis or on non-dialysis days - 2–10 min WU - From 1 to 3 sets × 8 to 15 repetitions - Exercise with resistance training equipment - Only lower body exercises using ankle weights - Isometric and isotonic exercises for the lower body and abdomen - Response Seated Leg Curl Thigh Extension pulley - Pneumatic leg press machine	- 1.5 s concentric phase, 0.5 s isometric and 3 s eccentric phase - 2 s concentric and 2 s eccentric - Not reported - Intensity 60–80% of 1-5RM, individual basis to 60–70% of HRMax, 40–60% of VO_2PEAK_ or 12–17 Borg’s scale	- 1-to-2-min rest between sets and exercises - 1 min between sets - Not reported	From 12 weeks to 1 year	From 2 to 5 times per week	From 30 to 110 min or not reported
	Control	No exercise or sham exercise						
Hu et al. [34]	Exercise	11 RT, 10 RT + AE	- During dialysis - From 3 to 5-min WU - From 1 to 3 sets × 8 to 20 repetitions - As many repetitions as possible - Repetitions until momentary failure occurred - From 4 to 11 types of exercises using elastic bands and soft weights - 10 sets of 10 repetitions lower limb raising - Exercises in both lower limbs and the contralateral arteriovenous fistula upper limb	- 1.5 s concentric phase, 0.5 s isometric and 3 s eccentric phase - Not reported - Intensity 60% of 1-3RM, 10–17 Borg’s scale or according to participant’s tolerance	- 1–2-min rest between each set - 2-to-3-min rest between each exercise - Rest according to the necessity of the participants - Not reported	From 8 weeks to 10 months	From 2 to 3 times per week	From 20 to 60 min or not reported
	Control	Usual care, sham exercise or no exercise						
Huang et al. [35]	Exercise	4 RT, 7 RT + AE	- Prior, after or during dialysis - 2-to-5-min WU - From 1 to 3 sets × 8 to 15 repetitions - 10 types of exercise with free-weight dumbbells, weighted ankle cuffs and elastic band tubing - Response Seated Leg Curl Thigh Extension pulley	- Not reported - Intensity 50–60% of the 1-3RM, 12–17 Borg’s scale or according to participant’s tolerance	- 60 s between sets - Not reported	From 8 weeks to 1 year	From 2 to 3 times per week	From 10 to 90 min or not reported
	Control	Usual care or sham exercise						
Lu et al. [36]	Exercise	10 RT, 4 RT + AE	- During dialysis - 5-to-10-min WU - From 1 to 2 sets × 8 to 20 repetitions - From 10 to 11 types of exercise - Exercises with grip ring, free weight dumbbells, ankle cuffs or leg press equipment - Training with chair stand exercise - Response Seated Leg Curl Thigh Extension pulley	- 10 s isometric contractions - 1.5 s concentric phase, 0.5 s isometric and 3 s eccentric phase - 5 to 10 s held isometric contractions - Standing up in 3 s and sitting down in 3 s - Intensity 50–80% of 1-5RM, 60% of individual’s maximal capacity, 12–17 Borg’s scale or not reported	- 1 to 2 min between sets and exercises - 2-min rest between sets - According to the necessity of the participants - Not reported	From 8 weeks to 5 months	From 2 to 3 times per week	From 15 to 60 min or not reported
	Control	Usual care, no exercise, sham exercise, attention care or placebo						
Matsuzawa et al. [37]	Exercise	13 RT, 6 RT + AE	- Prior, after or during dialysis - 5-min WU - From 1 to 3 sets × 10 to 15 repetitions - Exercises with a pneumatic leg press machine - Exercises for upper and lower body using elastic bands, sandbags and dumbbells - Response Seated Leg Curl Thigh Extension pulley	- 1.5 s concentric phase, 0.5 s isometric and 3 s eccentric phase - Not reported - Intensity 50–80% of 1-5RM, 60% of individual’s maximal capacity, 12–17 Borg’s scale or not reported	- 1 to 2 min between sets - 1 min between sets - Not reported	From 8 weeks to 12 months	From 2 to 3 times per week or not reported	From 20 to 90 min or not reported
	Control	Usual care or no exercise						
Molsted et al. [38]	Exercise	8 RT	- Prior, after or during dialysis - 5-min WU - From 1 to 3 sets × 10 to 15 repetitions - Exercises with a pneumatic leg press machine - Exercises for upper and lower body using elastic bands, sandbags and dumbbells - Response Seated Leg Curl Thigh Extension pulley	- 1.5 s concentric phase, 0.5 s isometric and 3 s eccentric phase - Not reported - Intensity 50–80% of 1-5RM or 15–17 Borg’s scale	- 1–2 min between sets - 1 min between sets - 2-min rest period between sets - Not reported	From 12 weeks to 6 months	3 times per week or not reported	30 min or not reported
	Control	Usual care or stretching						
Pu et al. [39]	Exercise	4 RT, 7 RT + AE	- Prior, after or during dialysis - 5-min WU - Isotonic and isometric exercises for the lower limb - Exercises for upper and lower body using elastic bands, sandbags and dumbbells	- 1.5 s concentric phase, 0.5 s isometric and 3 s eccentric phase Intensity 50 to 80% of 1–5 RM or 15–17 Borg’s scale - Not reported	- 1–2 min between sets - 1 min between sets	From 8 weeks to 12 months	From 2 to 3 times per week or not reported	From 10 to 90 min or not reported
	Control	No exercise						
Scapini et al. [40]	Exercise	5 RT, 10 RT + AE	- Prior to, after, during dialysis or on non-dialysis days - 5-to-10-min WU - From 2 to 3 sets × 8 to 15 repetitions - Isotonic and isometric exercises for the lower limb and abdomen - 4 lower limb exercises with ankle cuffs and elastic bands -5 upper limb exercises using dumbbells and 5 lower limb exercises using ankle weights - Close and open-chained exercises - Apparatus for leg extension and flexion - Response Seated Leg Curl Thigh Extension pulley	- Intensity 50–80% of the 1-5RM, 60–70% HRMax or 12–17 Borg’s scale	- 1 min rest between sets - Not reported	From 8 weeks to 1 year	From 2 to 4 times per week	From 20 to 90 min or not reported
	Control	No exercise or placebo						
Schardong et al. [41]	Exercise	8 EMS	- During dialysis - 5-min WU - Exercise with dual-channeled portable stimulators, a calibrated electrical stimulator or a neoprene garment	- From 1.5 to 20 s stimulation - Intensity according to patient’s tolerance or maximum painless level of stimulation	- From 0.75 to 20 s rest between stimulations - Rest time decreasing as the protocol advances (from 50 to 10 s) - Not reported	From 8 to 20 weeks	From 2 to 3 times per week	From 20 to 60 min or not reported
	Control	Control, placebo or exercise with no EMS						
Segura-Orti [42]	Exercise	2 RT, 4 RT + AE	- Before, after, during dialysis or on non-dialysis days - 2-to-10-min WU - From 1 to 3 series × 8 to 10 repetitions - 10 exercises for lower and upper body - 14 types of low resistance - Response Seated Leg Curl Thigh Extension pulley	- Not reported - Intensity 50–60% of the 3-5RM or Borg’s scale 15–17	- Not reported	From 12 to 24 weeks	From 2 to 4 times per week	From 30 to 90 min or not reported
	Control	No exercise or placebo						
Sheng et al. [43]	Exercise	7 RT, 4 RT + AE	- Prior, after or during dialysis - From 2 to 5-min WU - From 2 to 3 series × 8 to 15 repetitions - Lower body exercises using ankle weights - Upper and lower body exercises using elastic bands and sandbags - 10 types of exercises - isotonic quadriceps and hamstring exercises - Isotonic and isometric exercises for the lower limbs and abdomen - Response Seated Leg Curl Thigh Extension pulley	- 1.5 s concentric phase, 0.5 s isometric and 3 s eccentric phase - Intensity 40–60% of the 1-5RM, 65–85% of VO_2max_ or 11–17 Borg’s scale	- 60 s rest between sets - 1 to 2 min between sets - Not reported	From 8 weeks to 10 months	From 2 to 3 times per week	From 10 to 60 min or not reported
	Control	No exercise						
Wu et al. [44]	Exercise	12 RT + AE	- During dialysis or on non-dialysis days - 10-min WU - From 1 to 3 set × 8 to 30 repetitions - Exercises for a variety of upper and lower body muscle groups - Exercises with a handgrip strengthening device and mid-level load exercises - Exercises in fixed resistance machines	- Not reported - Intensity 70–80% of the 1RM, 12–14 Borg’s scale, 50–80% of heart rate reserve or not reported	- Not reported	From 12 weeks 1 year	- From 2 to 3 times per week - 6 times per month	From 60 to 120 min or not reported
	Control	Usual care and no exercise						
Zhang et al. [45]	Exercise	14 RT	- Prior to or during dialysis - 5-to-10-min WU - From 3 to 4 sets × 8–30 repetitions - Leg press exercises - 6 upper body and 6 lower body exercises with elastic bands and sandbags - 11 different types of exercises	- 1.5 s concentric phase, 0.5 s isometric and 3 s eccentric phase - Not reported - Intensity 70–80% of the 1-5RM, 12–17 Borg’s scale	-2-min rest period between sets - 1-to-2-min rest between sets - 1 min rest between sets - 3-min rest between exercises - Rest according to the necessity of the patient - Not reported	From 12 to 24 weeks	- From 2 to 3 times per week	From 30 to 50 min or not reported
	Control	No exercise or stretching						

*AE* aerobic exercise; *DPGE* dialysis patient’s guide exercise; *EMS* electrical myostimulation; *HRMAX* maximum heart rate; *MIP* maximum inspiratory pressure; *RDT* regular dialysis treatment; *RM* repetition maximum; *ROM* range of motion; *RPE* rate perceived exertion; *RT* resistance training; *WU* warm-up

### Methodological quality analysis

**Fig. 2:**
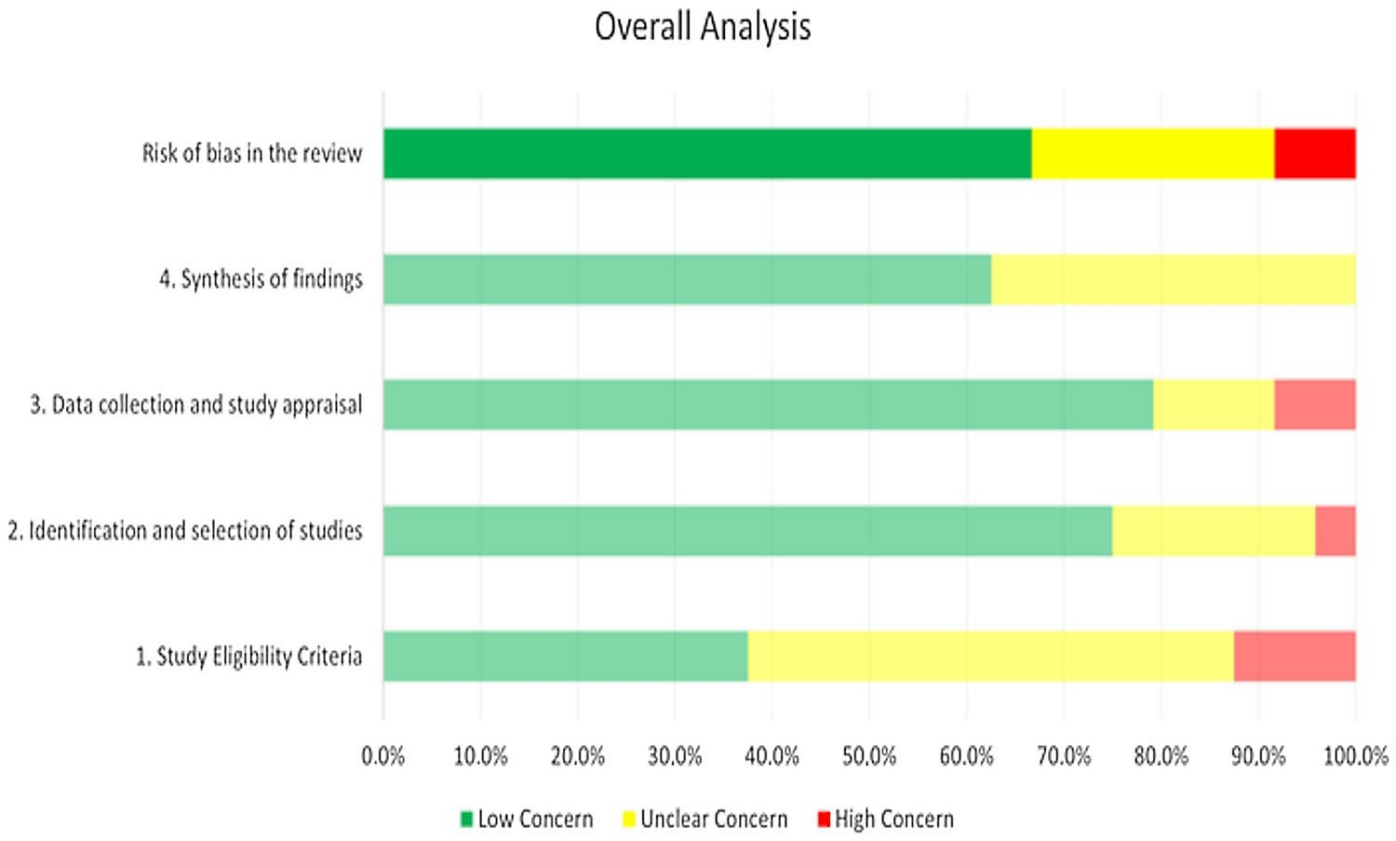
Summary plot graph of the ROBIS Scale

To assess the level of risk of bias, two independent reviewers used the ROBIS tool, and discrepancies were resolved by discussion and consensus. Figure 2 shows a summary plot graph summarizing the results obtained for each of the included reviews. Overall results showed that most of the studies had low risk of bias (67.5%). The risk of bias in the remaining studies was unclear (25%) or high (7.5%). The level of agreement between the reviewers in the assessment was high (κ = 0.820).

### Main findings

#### Functional capacity

**Fig. 3:**
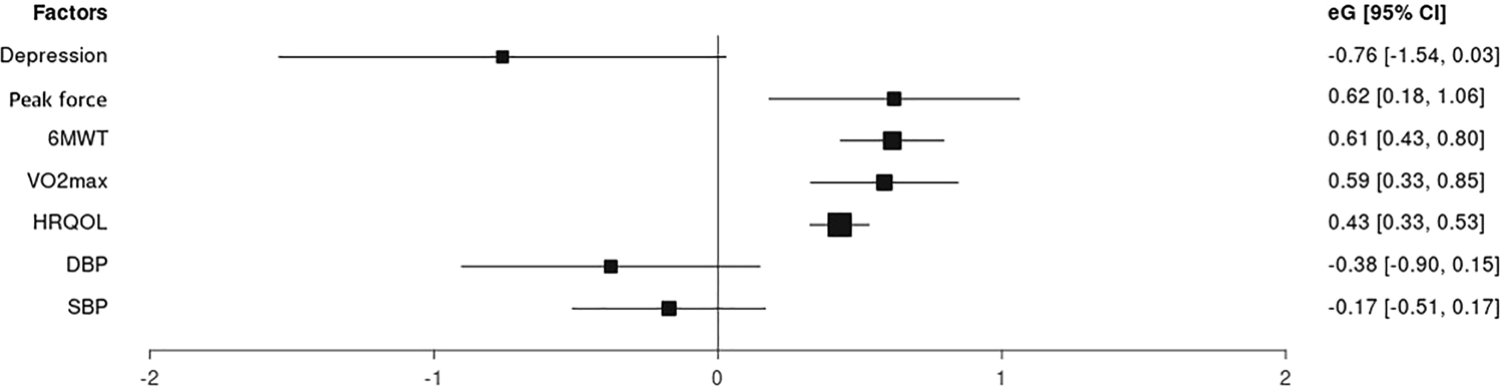
Forest plot

Nine studies (38%) [25, 26, 29, 30, 32, 36, 37, 41, 45] including 2290 participants reported effects of resistance training on functional capacity. Functional capacity was assessed in every study through the 6MWT. Overall effect size of resistance training with respect to functional capacity showed statistically significant differences with a moderate clinical effect (SMD = 0.614, CI 95% 0.433–0.795), and heterogeneity among the studies was high (*I*^2^ = 74.43%) (Fig. 3). The certainty of evidence was moderate, showing that resistance training increases functional capacity in patients with ESRD (Table 3).

**Table 3:** Summary of findings and quality of evidence according to Physical Activity Guidelines Advisory Committee Grading Criteria (PAGAC)

Systematic review research questions	Applicability	Generalizability	Risk of bias or study limitations	Quantity and consistency	Magnitude and precision of effect	Effect [95% CI]	Evidence
Depression	Strong	Strong	Limited	Limited	Strong	−0.76 [−1.54, 0.03]	Moderate
Peak force	Strong	Strong	Limited	Limited	Strong	0.62 [0.18, 1.06]	Moderate
6MWT	Strong	Strong	Limited	Limited	Strong	0.61 [0.43, 0.80]	Moderate
VO_2_ max	Strong	Strong	Limited	Limited	Strong	0.59 [0.33, 0.85]	Moderate
HRQOL	Strong	Strong	Limited	Limited	Strong	0.43 [0.33, 0.53]	Moderate
DBP	Strong	Strong	Limited	Limited	Strong	−0.38 [−0.90, 0.15]	Moderate
SBP	Strong	Strong	Limited	Limited	Strong	−0.17 [−0.51, 0.17]	Moderate

95% CI: 95% Confidence interval; HRQOL: Health Related Quality of Life; 6MWT: 6 Minute Walking Test; SBP: Systolic Blood Pressure; DBP: Diastolic blood pressure; VO_2_ max: Maximal oxygen uptake

#### Aerobic capacity

Twelve studies (50%) [24, 26, 28, 30, 32, 33, 35, 37, 39, 40, 42, 43] including 4037 participants reported the effects of resistance training on aerobic capacity. Every included study assessed aerobic capacity through the VO_2max_. Overall effect size of resistance training with respect to aerobic capacity showed statistically significant results with small clinical effects (SMD = 0.587, CI 95% 0.328–0.845) and high heterogeneity among the studies (*I*^2^ = 89.82%) (Fig. 3). The certainty of evidence was moderate, showing that resistance training increases aerobic capacity in patients with ESRD (Table 3).

#### Peak force

Six studies (25%) [27, 32, 33, 36, 37, 41] including 2160 participants reported the effects of resistance training on peak force. Every included study assessed peak force through dynamometry. Overall effect size of resistance training with respect to muscle strength showed statistically significant differences with moderate clinical effects (SMD = 0.621, CI 95% 0.181–1.061) and heterogeneity among the studies was very high (*I*^2^ = 90.752%) (Fig. 3). The certainty of evidence was moderate, showing that resistance training increases muscle strength in patients with ESRD (Table 3).

#### Depression

Five studies (21%) [25, 28, 31, 32, 39] including 960 participants reported the effects of resistance training on depression symptoms. Depression was assessed through the Beck Depression Inventory. Overall effect size of resistance training with respect to depression did not show statistically significant differences (SMD = −0.759, CI 95% −1.545–0.027) and heterogeneity among the studies was high (*I*^2^ = 93.75%) (Fig. 3). The certainty of evidence was moderate, showing that resistance training does not decrease depression in patients with ESRD (Table 3).

#### Health-related quality of life

Ten studies (42%) [25, 27, 28, 32, 34, 37–39, 42, 43] including 2481 participants assessed the effects of resistance training on HRQOL, which was assessed using the Short-Form 36 questionnaire (SF-36), specifically with its Physical Component Scale. Overall effect size of resistance training with respect to HRQOL showed statistically significant differences with small clinical effects (SMD = 0.429, CI 95% 0.326–0.532) and small levels of heterogeneity among the studies (*I*^2^ = 36.09%) (Fig. 3). The certainty of evidence was moderate, showing that resistance training increases HRQOL in patients with ESRD (Table 3).

#### Systolic and diastolic blood pressures

Four studies (17%) [30, 33, 39, 40] including 1067 participants reported effects of resistance training on systolic blood pressure and diastolic blood pressure. Changes were assessed by analyzing the change in values in mmHg for both systolic and diastolic blood pressures. Overall effect size of resistance training with respect to systolic blood pressure did not show statistically significant differences (SMD = −0.173, CI 95% −0.512–0.167) and heterogeneity among the studies was high (*I*^2^ = 82.61%). Overall effect size of resistance training with respect to diastolic blood pressure did not show statistically significant differences (SMD = −0.377, CI 95% −0.902–0.147) and heterogeneity among the studies was high (*I*^2^ = 88.17%) (Fig. 3). The certainty of evidence was moderate, showing that resistance training does not influence systolic and diastolic blood pressures in patients with ESRD (Table 3).

### Overlapping analysis

**Fig. 4:**
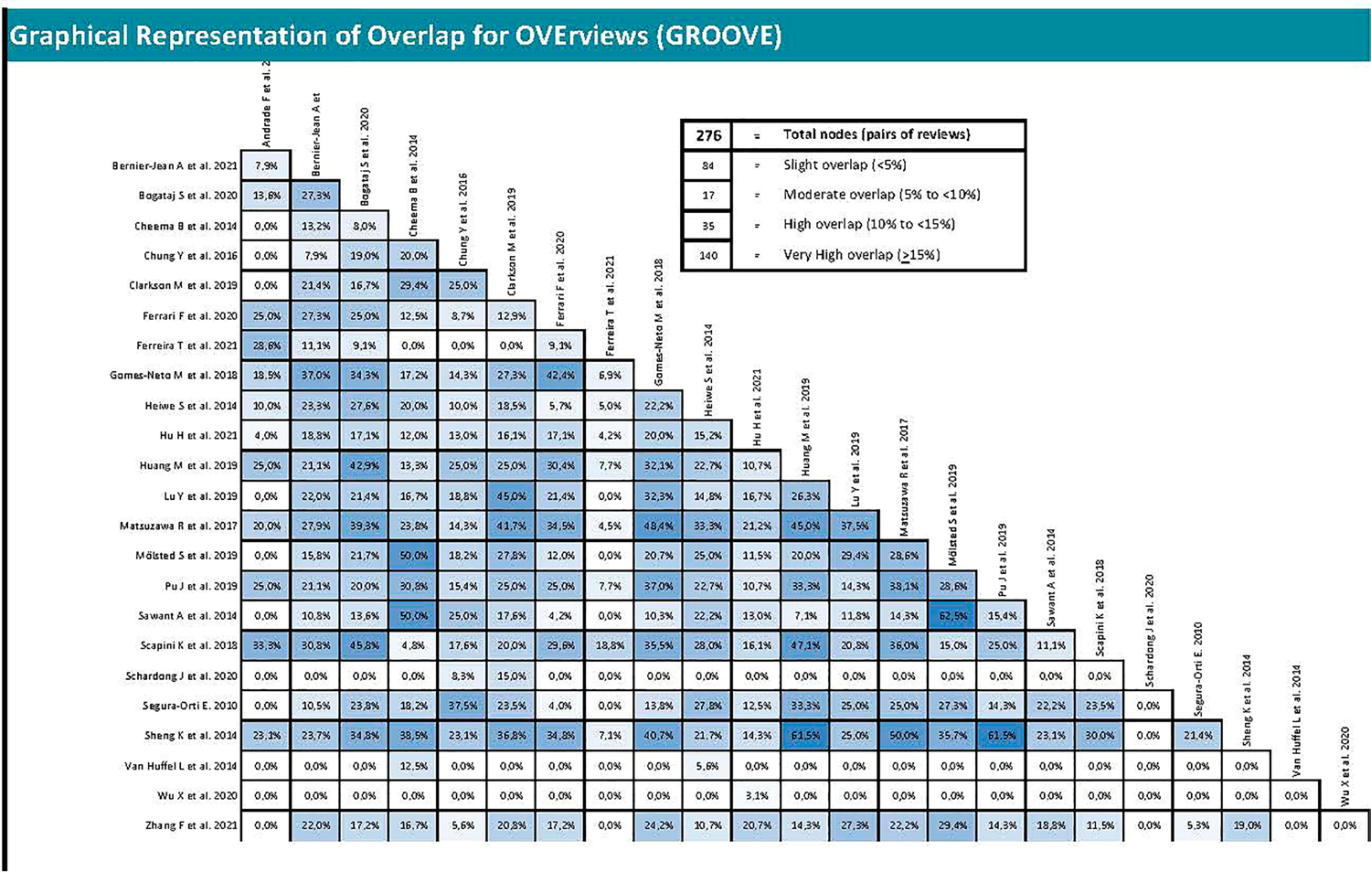
Graphical representation of overlap for Overviews

The overlap analysis performed using the GROOVE tool revealed a total corrected overlap area of 9.45%, showing a moderate level of overlap. The detail of the overlap is shown in Fig. 4.

**Fig. 5:**
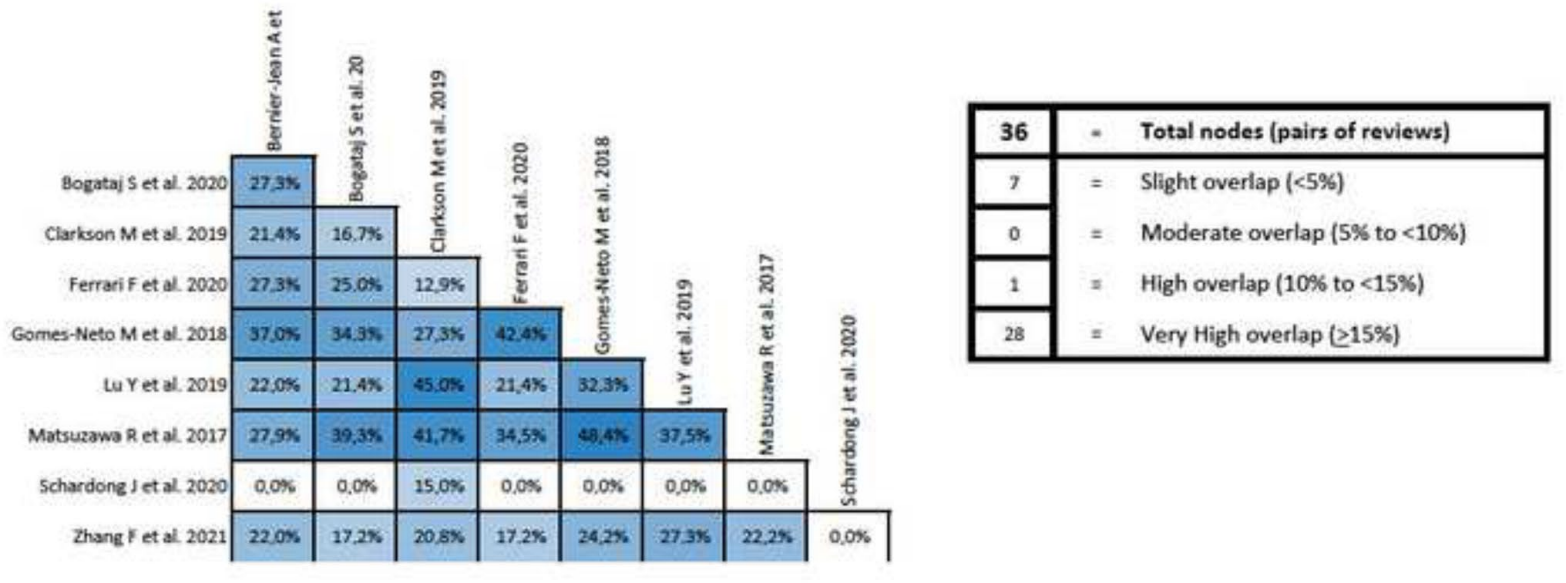
Overlap analysis for the 6MWT

**Fig. 6:**
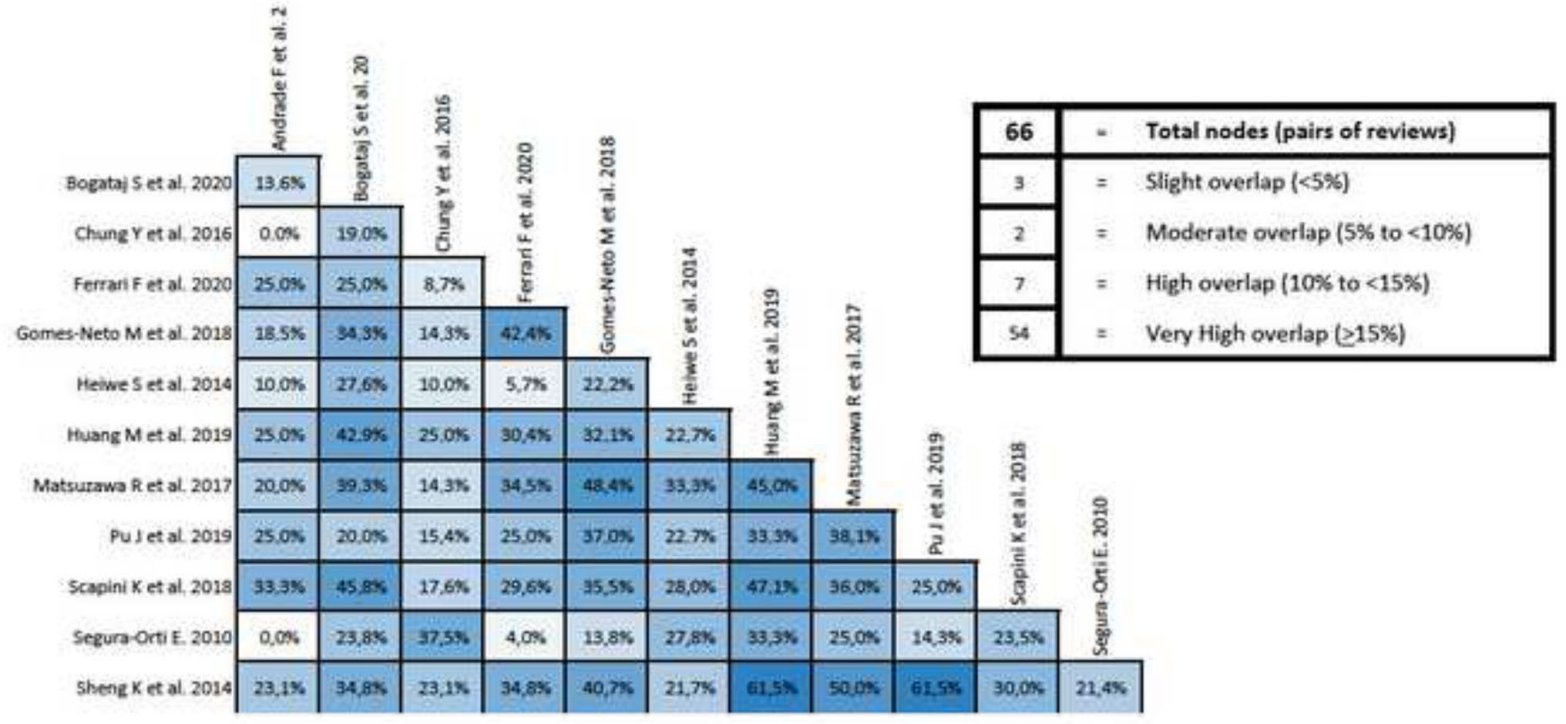
Overlap analysis for the VO_2max_

**Fig. 7:**
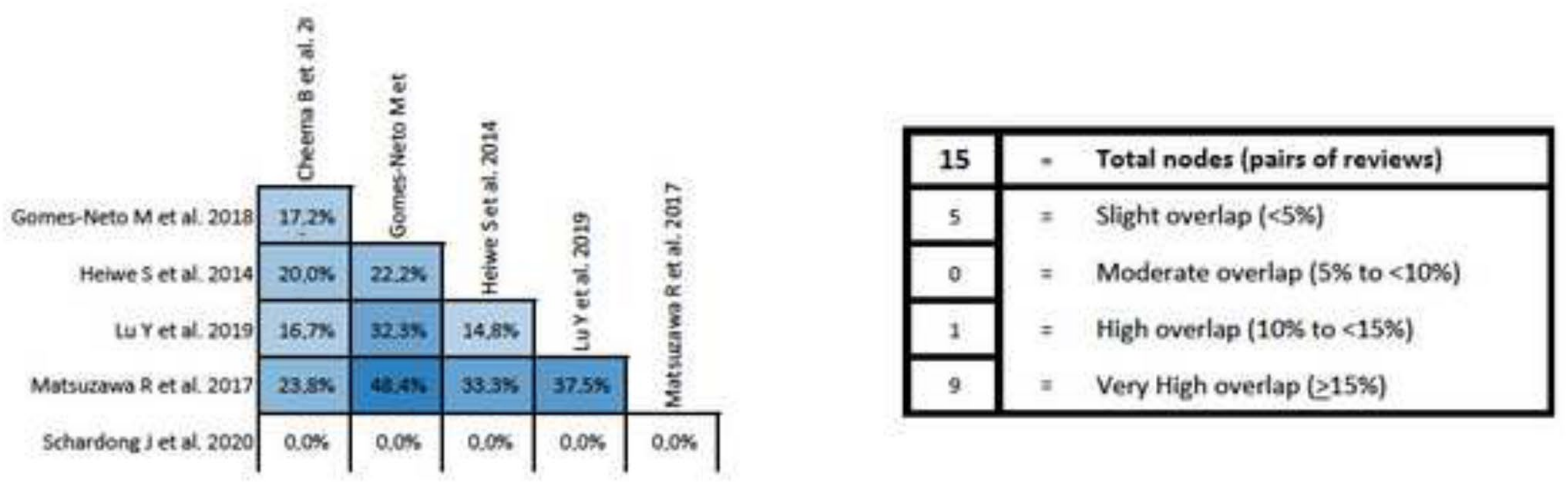
Overlap analysis for peak force

**Fig. 8:**
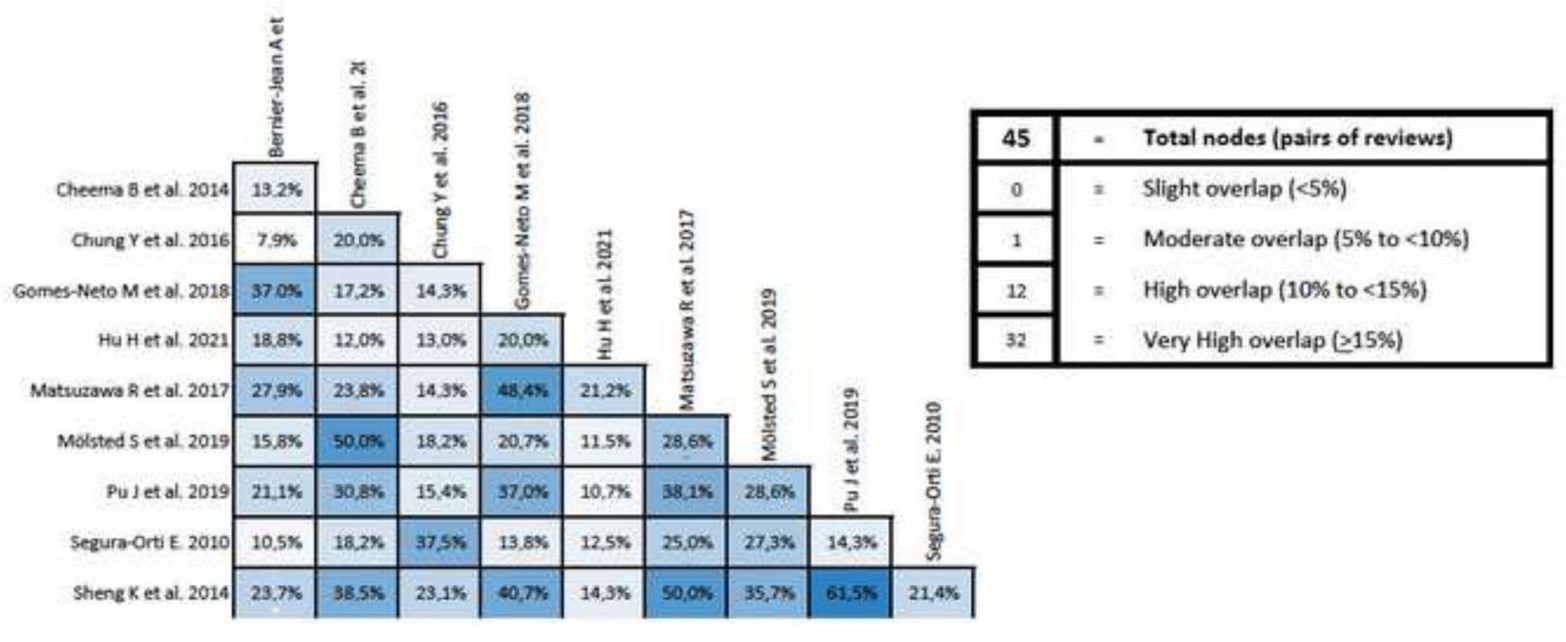
Overlap analysis for the HRQOL

**Fig. 9:**
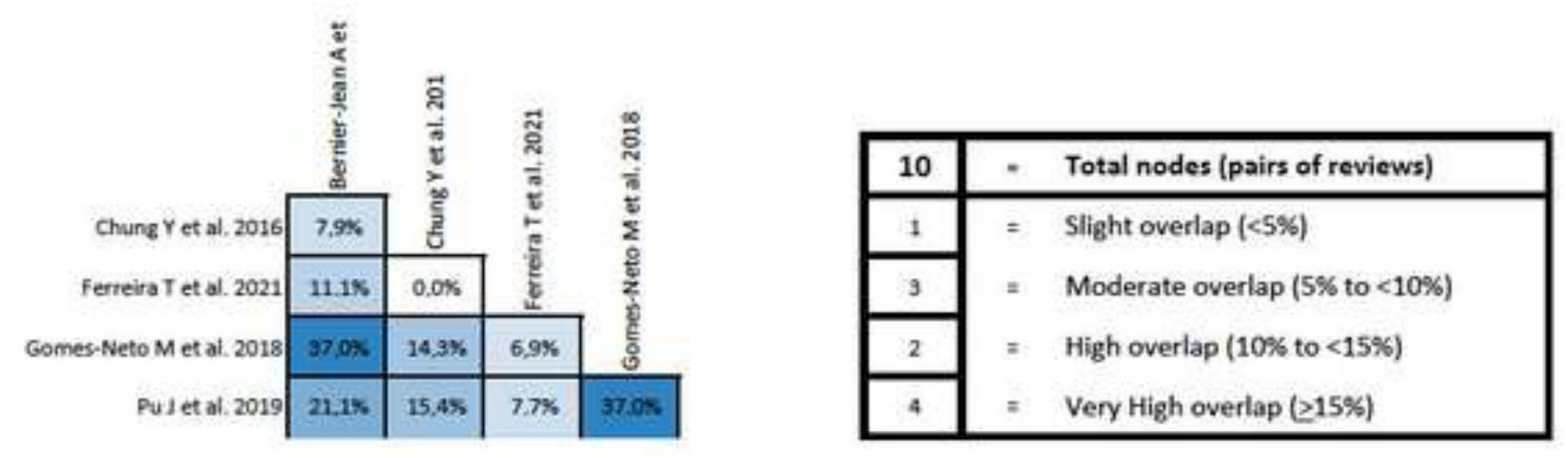
Overlap analysis for depression

**Fig. 10:**
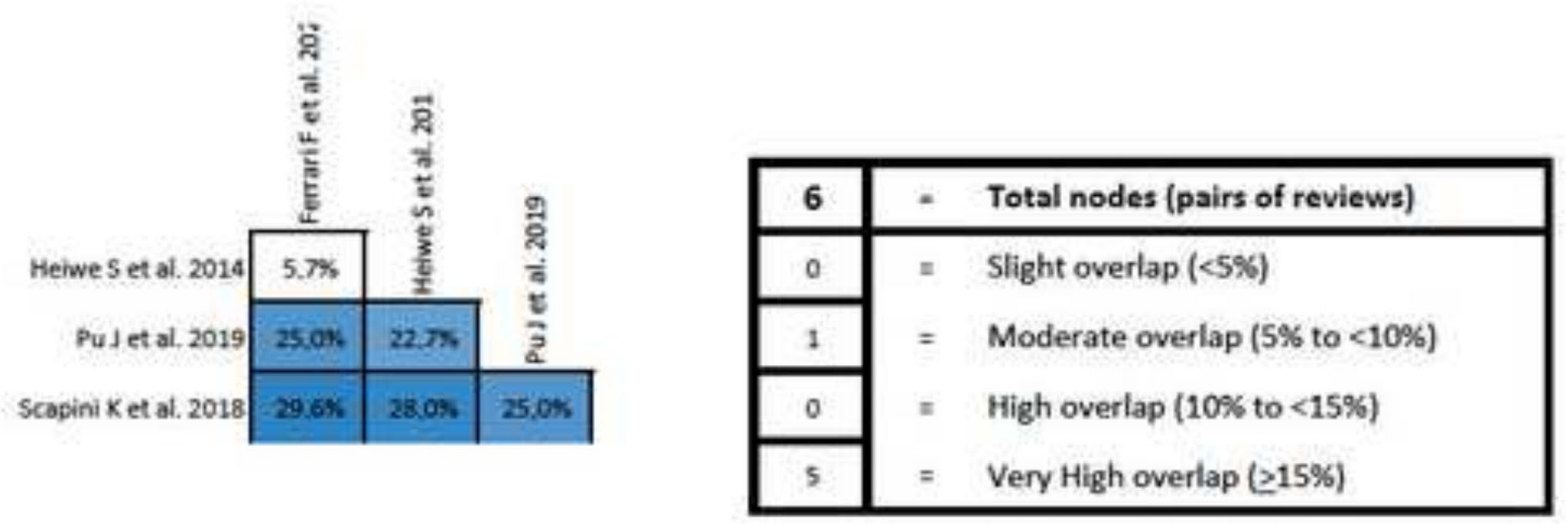
Overlap analysis for the SBP and DBP

In addition, an overlap analysis was performed for each variable. In the 6MWT, a total of nine reviews including 70 different primary studies were included, which represented a corrected covered area of 18.39% (very high overlap) (Fig. 5). Regarding VO_2max_, 12 reviews were included with 165 different primary studies, representing a corrected covered area of 19.21% (very high overlap) (Fig. 6). Similar results were found in peak force outcome, including a total of six reviews, 51 primary studies and a corrected covered area of 16.08% (very high overlap) (Fig. 7). In relation to HRQOL, a total of 10 reviews and 63 different primary studies were included, representing a corrected covered area of 15.34% (very high overlap) (Fig. 8). Regarding the depression variable, five reviews and 47 primary studies were included, representing a corrected covered area of 18.62% (very high overlap) (Fig. 9). Finally, in SBP and DBP, four reviews and 39 primary studies were included, representing a corrected covered area of 19.66% (very high overlap) (Fig. 10).

## Discussion

This umbrella review analyzed the benefits and the effects of resistance training in patients with ESRD undergoing dialysis and assessed the quality of the published literature. Multiple outcomes that relate to patient’s overall well-being were analyzed, and positive effects were found in functional capacity, aerobic capacity, peak force and HRQOL. Twenty-four studies were eventually included in the review, presenting a substantial quantity of literature evidence that outlines the benefits of resistance training in patients undergoing dialysis.

Physical factors, such as functional capacity, aerobic capacity, and peak force, as well as psychosocial factors, such as HRQOL, showed positive results, reinforcing the idea that in this usually sedentary population, exercise interventions have an effect on several dimensions that intervene in the biopsychosocial well-being of these patients. Nonetheless, some of them showed small clinical effects, but still had a considerable margin of improvement, so developing adapted exercise interventions that are controlled and that follow a specific protocol for patients on dialysis could potentially result in even greater improvements.

Both functional and aerobic capacities, assessed through the 6MWT and VO_2max_, respectively, showed positive results. These assessments are widely used in the literature and are closely correlated [36], therefore it is logical to assume that an improvement in one will potentially generate improvement in the other. Because functional capacity showed bigger differences than aerobic capacity, the inclusion of aerobic exercise interventions would be relevant to more specifically improve this outcome [46].

HRQOL also showed positive effects, albeit with a small clinical effect. Functional impairment also contributes to impaired HRQOL [27], therefore, an improvement in functional outcomes could also imply an improvement in HRQOL, as shown in this review. Even though there is no standard definition of HRQOL, it is widely accepted that it includes physical, psychological and social domains of life [34], and therefore an improvement in this outcome could be more limited solely by performing exercise.

Significant differences were found regarding muscle peak force, with a moderate effect. The moderate effect might be due to insufficient stimulus in the training programs, that is unable to produce enough improvement. Results presenting smaller effects could require more complete programs that include sufficient exercise intensity in the interventions, together with adequate exercise volume (quantity of exercise measured in repetitions and sets), because of the overcautious approach when applying exercise in a frail population.

Additionally, these results might be influenced by other factors involved in muscle performance, such as dietary restrictions and metabolic inflammation. Even though resistance training is considered a potent insulin-like growth factor inducer [36], restrictions in protein intake, along with enhanced proteinuria, limit the synthesis of proteins in patients with ESRD, especially in those with associated prevalent metabolic comorbidities, such as diabetes mellitus [47]. Also, the associated inflammatory environment could create abnormalities in muscle fibers due to cellular adaptations, including enzymatic and contractile protein changes, also limiting protein synthesis [41] and impairing muscle performance.

No statistically significant effects were found in depression outcomes. This might be due to the biopsychosocial nature of depression, and the necessity to deal with this problem from a more multidisciplinary approach, including psychotherapy, for instance. Also, results in this analysis show high variability, meaning that, even though depression has high prevalence rates in this population [31, 32], patients on dialysis might not suffer from it, hence not perceiving the benefits of resistance training in this outcome.

No statistically significant changes were found in either systolic or diastolic blood pressure levels. Our results are consistent with those found by Heiwe et al. [33]. Even though intradialytic exercise interventions have been proposed to improve blood pressure levels [30, 39], these results should be cautiously interpreted because these reviews do not isolate different types of exercise interventions, so their conclusions are not strictly based on resistance training interventions alone. Also, resistance training protocols that elicit the most favorable blood pressure level benefits remain elusive, and populations taking antihypertensive medications, such as patients with ESRD, show smaller reductions in blood pressure levels [48]. In patients on hemodialysis, hypertension is closely related to overload, caused by retention due both to the dialytic treatment and the disease’s pathophysiology (uremic state, anemia, electrolyte imbalance) [24]. The authors of some studies even suggest that fluid overload (> 5% above dialysis body weight) must be considered an exclusion criterion, as they deem it makes patients unable to exercise during dialysis sessions [25].

Risk of bias was assessed with the ROBIS tool, a domain-based resource supported by signaling questions. Results from this analysis showed that most studies (63%) included had low risk of bias levels. However, it is important to outline that the ROBIS tool is not a fully objective assessment resource, and some of the items that the tool includes depend on the examiner’s interpretation, so careful conclusions must be extracted from this analysis.

The overlap analysis performed using the GROOVE tool showed a total moderate level of overlap. However, when analyzing the overlap for each individual outcome, high overlapping levels were observed in every outcome (6MWT (18.39% of overlap), VO_2max_ (19.21% of overlap), peak force (16.08% of overlap), HRQOL (15.34% of overlap), Depression (18.62% of overlap), and SBP and DBP (19.66% of overlap)). When conducting clinical trials implementing exercise interventions in participants with ESRD, throughout the literature it is common to assess the same outcomes through the same assessment methods, and this increases the chances that the same clinical trial will be included in several reviews. This could potentially create an overpowering of the results in a specific outcome, therefore creating a bias that might lead to false assumptions about the results. However, overlap can also be considered an advantage as it simplifies the meta-reviewer’s work and reinforces the conclusions that can be extracted [49]. The assessment of study overlapping is important, as systematic reviews must have well-defined search strategies and should be conducted only when necessary, avoiding repetitive reviews that might lead to unreliable and overpowered clinical results.

Our study shows several strengths. There is a considerable number of included studies, and these show appropriate methodological quality levels. Studies included in the review were consistent with the assessment method choice when analyzing every outcome of this review (functional capacity was assessed with the 6MWT, aerobic capacity with the VO_2max_ and so on), facilitating further analysis. Finally, even though resistance training programs were inconsistent due to the lack of following a standardized developed protocol, the included exercise programs were able to show positive results, implying that even more positive results can be potentially achieved if proper strength training protocols are followed.

Finally, this review also presents limitations. Even though the research was conducted in several scientific databases and other resources were also consulted, not every existing scientific database was accessed, meaning that not every study assessing resistance training in patients undergoing dialysis might have been included. Additionally, clinical characteristics of the participants enrolled in the exercise programs, detailed descriptions of the exercise programs used, and adherence rates were not thoroughly reported or should be carefully interpreted, and this may have influenced the interpretation of the results. For instance, even though we believe that this does not have a significant effect on the results, one of the studies included in this review [27] considered a study in pre-dialysis patients.

We believe that the meta-analytic results in this study reinforce the idea of recommending resistance training exercise interventions as part of the daily management of patients undergoing hemodialysis, due to the effectiveness in improving biopsychosocial dimensions of this disease. Future studies should focus on thoroughly reporting exercise parameters to determine the optimal loading.

## Data Availability

The datasets generated and analyzed during the current study are available from the corresponding author on reasonable request.
